# Editorial: Updates on tuberculosis control and management

**DOI:** 10.3389/fpubh.2022.1126429

**Published:** 2023-01-06

**Authors:** Li Cai, Xinyi Hu, Yadong Huang, Xibao Huang, Yeqing Tong

**Affiliations:** ^1^Department of Infectious Diseases, Wuhan Center for Disease Control and Prevention, Wuhan, China; ^2^School of Public Health, Wuhan University, Wuhan, China; ^3^Global Study Institute, University of Geneva, Geneva, Switzerland; ^4^Department of Hospital Infection Control, Wuhan No.1 Hospital, Wuhan, China; ^5^Department of Infectious Diseases, Hubei Center for Disease Control and Prevention, Wuhan, China

**Keywords:** updates, tuberculosis, control, prevention, management

Tuberculosis (TB), a potentially serious infectious disease caused by a bacterium called *Mycobacterium tuberculosis* (*M. tuberculosis*), is a major cause of ill health and death worldwide. The World Health Organization (WHO) strategy to end TB was adopted in 2014 by the 67th World Health Assembly and aligned in 2015 as part of the ambitious Sustainable Development Goals (SDGs) to end the global epidemic of tuberculosis by 2030. The WHO strategies include milestones and targets working on easing the burden of disease by 2030 and 2035, with three remarking indicators on TB incidence, death, and costs induced by disease, respectively. Global goals provided a comprehensive framework for each region and country to localize their actions. Seven years after the launch of SDGs, according to the global tuberculosis report 2022, the world is not on track to achieve the End TB targets either globally or in most WHO regions and countries. Some reversals of progress can be observed during the pandemic of COVID-19 as disclosed in WHO Global Tuberculosis Report (1), Guidelines for Tuberculosis Control in New Zealand (2), and Major infectious disease (3). Under such circumstances, the application of new technologies and the iteration of case management methods are crucial in catching up with the goal. This editorial summarized key findings from four papers on the prevention and control of TB cases.

The COVID-19 pandemic diverted global attention to the End TB targets (4). WHO report revealed a large drop in TB case notifications and death during the COVID-19 pandemic, suggesting an increased number of undiagnosed, untreated, and even died with no active tuberculosis record. A decline from both the supply and demand side of diagnosis and treatment could explain potential reasons for the drop. Redirected to COVID-19, healthcare resources ready for tuberculosis became insufficient. At the same time, healthcare demand for tuberculosis plummeted due to physical distancing measures, patients' stigma associated with its symptoms similar to COVID-19, etc.

The second challenges are its highly-structured disease burden for the disadvantaged (5). Socio-economic factors attributed to TB burden include malnutrition, low body mass index, poverty, and income per capita, etc. (6). The COVID-19 pandemic further exacerbated this imbalance. Not only the direct healthcare cost but also indirect costs, including cost of hospital visit, medication, and potential opportunity cost of losing job, increased. Restrictions on mobility expose segments of the population, such as the homeless, immigrants, and incarcerated people, to the risk of contracting and spreading tuberculosis.

Growth in the number of people with undiagnosed and untreated TB called for action on the research and development of TB diagnosis and treatment. Diagnosis includes systematic screening on *Mycobacterium tuberculosis* complex (*M. tuberculosis* complex) and drug resistance of contacts and the high risk. Sputum smear microscopy, a technique dating to the 1880s, remained a primary tool for laboratory diagnosis for its convenience. Whereas, it falls short in terms of its timeliness and test sensitivity (7). The test sensitivity rate for the smear microscopy is especially low for children and the HIV-positive group (8). To overcome those limitations, Xpert MTB/RIF and its newest version Xpert Ultra, were developed and endorsed by WHO as a diagnostic tool for its faster and more accurate diagnosis that detects *M. tuberculosis* and rifampicin resistance simultaneously (9, 10). In addition to being prompt and precise, its fully-integrated system GeneXpert^®^ made extraction, amplification, and detection all-in-one with a user-friendly design (11). There were also specific technologies updated to improve the performance of tuberculosis induced-disease diagnosis. For instance, tuberculosis empyema remains common in many high disease burden countries and regions. A retrospective study conducted by Liu et al. examined the performance of TB-LAMP (loop-mediated isothermal amplification) by samples obtained from Shenyang Tenth People's Hospital patients' records from Jan 2017 to Jun 2021. The study demonstrated the potential of TB-LAMP in identifying TB empyema cases for its high sensitivity and specificity. In all, the technological diffusion across the global economy provides a prerequisite for novel technologies to be localized and applied to those high disease burden developing notions.

Tuberculosis preventive therapies (TPT), given to people at high risk of progressing from TB infection to disease, were highly recommended by the WHO as a critical public health measure to protect high-risk communities. Two major challenges, encountered for its application, are its restrictions on patients' inclusion (should exclude active cases) (12) and an overall low adherence rate for people to finish the 6 to 9 months TPT regimes (13). Min et al. initiated a Korean national cohort study to assess clinically meaningful factors associated with each step of the latent tuberculosis infection cascade of care (namely, test, diagnosis, clinics visit, treatment initiation, and treatment completion) in three congregate settings. The study indicated that age, treatment center, and initial treatment regimen were significant determinants of losses to care along the cascade of care. National research provided a big sample size to output high reference value implications for public health experts to improve policy designs for unnecessary loss to care. Some scholars explored complementary approaches to TPT prevention. A case-control study that enrolled 70 tuberculosis patients, as well as 210 matched controls examined the potential role of Vitamin D on the Han population. However, no significant difference has been found in the serum vitamin D level between the two groups. The evidence disclosed by Cai et al. indicated Vitamin D might be an independent risk factor for TB in the Han population. Vitamin D supplementation could be not only a protective factor but also a risk factor for different groups. Therefore, population-based vitamin D supplementation might not be widely-applied to the general public. E-health may be a solution that drives accurate identification of high-risk groups and can improve overall TPT adherence.

In terms of treatment, rifampicin and isoniazid are the most potent drugs for TB patients. Both Multidrug-resistant TB (MDR-TB) and rifampicin-resistant TB (RR-TB) require treatment with second-line drugs (14). The proportions of patients that are MDR-TB or RR-TB remain stable for a long time, but significant growth could be observed in 2021. Research and development for effective second-line treatment regimes should be accelerated (15). A retrospective study by Li et al. that examining potential factors associated with treatment failure among TB patients who were bacteriological confirmed to be resistant to at least one of the first-line drugs in Guangzhou Chest Hospital, China. The study concluded that being old and having pulmonary cavities were risk factors contributing to treatment failure. Also, patients not registered in Guangzhou and need to seek healthcare service across provinces and cities were risk factors associated with loss of follow-up along the cascade of care. The role of policy advocacy and finance should focus on the 4A (Acceptability, Affordability, Accessibility, and Awareness)'s of treatment regimes.

In general, SDG to end TB not only made an overarching blueprint for the whole world to put efforts in. Detailed strategies, comprising guidelines from diagnosis, prevention to treatment, provided a prerequisite for regions and countries, especially high disease burden countries, to localize actions. Identifying challenges brought up by the COVID-19 pandemic impinged on TB prevention and control, we also notice great opportunities for a more affordable and available TB healthcare.

## Author contributions

Conceived and designed the experiments: LC, YT, and XHu. Performed the experiments: YT, LC, YH, XHu, and XHua. Analyzed the data and wrote the paper: YT, XHu, and LC. Contributed reagents, materials, and analysis tools: YT, XHu, YH, and XHua. All authors contributed to the article and approved the submitted version.
